# Blood pressure estimation based on pulse rate variation in a certain period

**DOI:** 10.1038/s41598-020-58367-y

**Published:** 2020-01-29

**Authors:** Toshiyuki Hayase

**Affiliations:** Institute of Fluid Science, Tohoku University, 2-1-1 Katahira, Sendai, 980-8577 Japan

**Keywords:** Cardiovascular diseases, Diagnosis

## Abstract

Availability of daily continuous blood pressure (DCBP) has a strong impact to realization of healthy society. However, existing methods to obtain blood pressure of cuff type and cuff-less types utilizing correlation with pulse waveform, pulse transit time or pulse rate; or computation of circulation model are not suitable to obtain DCBP. Here we implemented a method based on a simple circulatory system model using pulse rate measurement to overcome the limitations, and showed that it provides appropriate estimation of DCBP. The present model consists of a circulatory dynamic system model and an inverse model of a circulatory control system with input of pulse rate and six model parameters representing standard pulse rate, elasticity of systemic arteries, peripheral vascular resistance, and characteristics of resistance and stroke volume control. Validity of the DCBP estimation method was examined by preliminary experiment for one subject in four days and that for four subjects in one day. DCBP estimation was performed with 24-hour pulse rate measurement by a wearable device and sphygmomanometer measurement for parameter determination and verification. Mean absolute errors in systolic/diastolic pressures were appropriate ones for preliminary experiments with 9.4/6.4 mmHg in four days and 7.3/5.9 mmHg in five subjects.

## Introduction

Availability of daily continuous blood pressure (DCBP) has a strong impact to realization of healthy society^1–4^. However, existing methods to obtain blood pressure of cuff type^5–7^ and cuff-less types utilizing correlation with pulse waveform^8–11^, pulse transit time^12–18^ or pulse rate^19^; or computation of circulation model^20,21^ are not suitable to obtain DCBP since repeated pressure by cuff or continuous measurement of pulse transit time cause uncomfortableness, body motion causes error in pulse waveform measurement, the correlation with pulse rate is not significant enough, and determination of circulation model parameters is difficult. In this study we implement a method based on a simple circulatory system model using pulse rate measurement to overcome the limitations, and show that it provides appropriate estimation of DCBP. The present model consists of a circulatory dynamic system model and an inverse model of a circulatory control system with input of pulse rate and six model parameters. Computation was performed with the model using 24-hour pulse rate measurements for one subject in four days and for four subjects in one day to obtain the optimum parameters and then to verify the proposed method.

**Figure 1:**
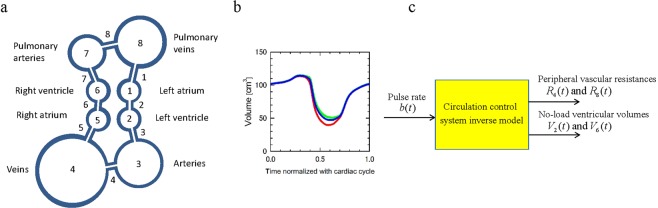
Circulatory system model consisting of circulatory dynamics model and circulatory control inverse model. **(a)** Circulatory dynamics model consisting of eight elastic containers (circles) representing left atrial (1), left ventricle (2), systemic arteries (3), systemic veins (4), right atrial (5), right ventricle (6), pulmonary arteries (7), and pulmonary veins (8); and eight liner resistors (line segments) connecting these containers. (**b)** Variation of ventricular volume by the reference^23^(green), and those at zero pressure, or no-load ventricular volumes, derived from the reference values for left ventricle (red) and right ventricle (blue). (**c)** Block diagram of circulatory control inverse model with input of pulse rate $b(t)$ and outputs of peripheral vascular resistance of systemic and pulmonary arteries, ${R}_{4}(t)$ and ${R}_{8}(t)$, and no-load ventricular volumes, ${V}_{2}(t)$ and ${V}_{6}(t)$.

The circulatory system model of this study consists of the circulatory dynamics model and the circulatory control inverse model. The real circulatory dynamics is a very complex fluid-structure coupled system^21^, but the present study models it as a simple lumped parameter dynamical system consisting of eight elastic containers representing a left atrial (1), a left ventricle (2), systemic arteries (3), systemic veins (4), a right atrial (5), a right ventricle (6), pulmonary arteries (7), and pulmonary veins (8); and eight liner resistors connecting these containers (Fig. 1a). The model can be described as basic equations of elastic mechanics and fluid dynamics, Windkessel model^22^, with variables of pressures and volumes of the containers ${P}_{i}(t)$ and ${V}_{i}(t)$, and flow rates between the containers ${Q}_{i}(t)$. Blood flow circulation is generated by introducing the variation of the ventricular volume at zero pressure, or *no-load ventricular volume*, based on the reference^23^ to the left and right ventricles (Fig. 1b). Parameters affecting the systemic arterial blood pressure ${P}_{3}(t)$ in this circulatory dynamics model is the standard pulse rate ${b}_{0}$, the elasticity ${E}_{30}$ and the peripheral resistance coefficient ${R}_{40}$ of the lumped systemic arteries at the standard pulse rate.

The real circulatory control system is a very complex one including the short term regulation by the autonomic nervous system and the long term hormonal regulation, etc.^24^, but the present study models it as a simple dynamical model with the input of pulse rate $b(t)$ and the outputs of peripheral vascular resistance of the systemic and pulmonary arteries, ${R}_{4}(t)$ and ${R}_{8}(t)$, and the no-load ventricular volumes, ${V}_{2}(t)$ and ${V}_{6}(t)$ (Fig. 1c). The present model represents an inverse system of the real circulatory control system in which the pulse rate is also an output. The circulatory control inverse model is constructed based on the following characteristics: (1) the circulatory control system maintains blood pressure constant, (2) baroreceptors have differential characteristics to effectively respond to short-term changes of blood pressure^25^. (3) the ventricular stroke volume increases with increase of the pulse rate^26^. Parameters of the circulatory control inverse model are the rate ${s}_{a}$ of variation of ventricular stroke volumes against the change of the pulse rate (cf. characteristic 3), the rate *s*_*r*_ of variation of peripheral vascular resistances against the change of the low frequency component of the pulse rate (cf. characteristic 1), and the time constant ${T}_{c}$ of the low pass filter characteristics of the control system with the input of the pulse rate (cf. characteristic 2). A total of six parameters of the present circulatory dynamics and circulatory control inverse models are determined by comparing the measured and calculated blood pressure values.

As the input of the present model, the pulse rate was measured for a male volunteer of 60 s (subject 1) by a commercially available wearable device with the measurement interval of one second in four days with random intervals of 27 months, 3 days, and 3 months. The subject 1 does not smoke. As the purpose of comparison, systolic and diastolic pressures and pulse rate were also measured in sitting position with an automatic sphygmomanometer in the above-mentioned four days with the interval of 30 min (wake up hours) or 60 min (sleeping hours). Another experiment was performed for three male volunteers of 20 s and one male volunteer of 40 s in the same way excepting that sphygmomanometer measurement was performed during only wake up hours in one day. These experiments were performed under the approval of the Ethics Committee of Tohoku University.

## Results

### Daily continuous blood pressure (DCBP) estimation for subject 1 in day 1

**Figure 2:**
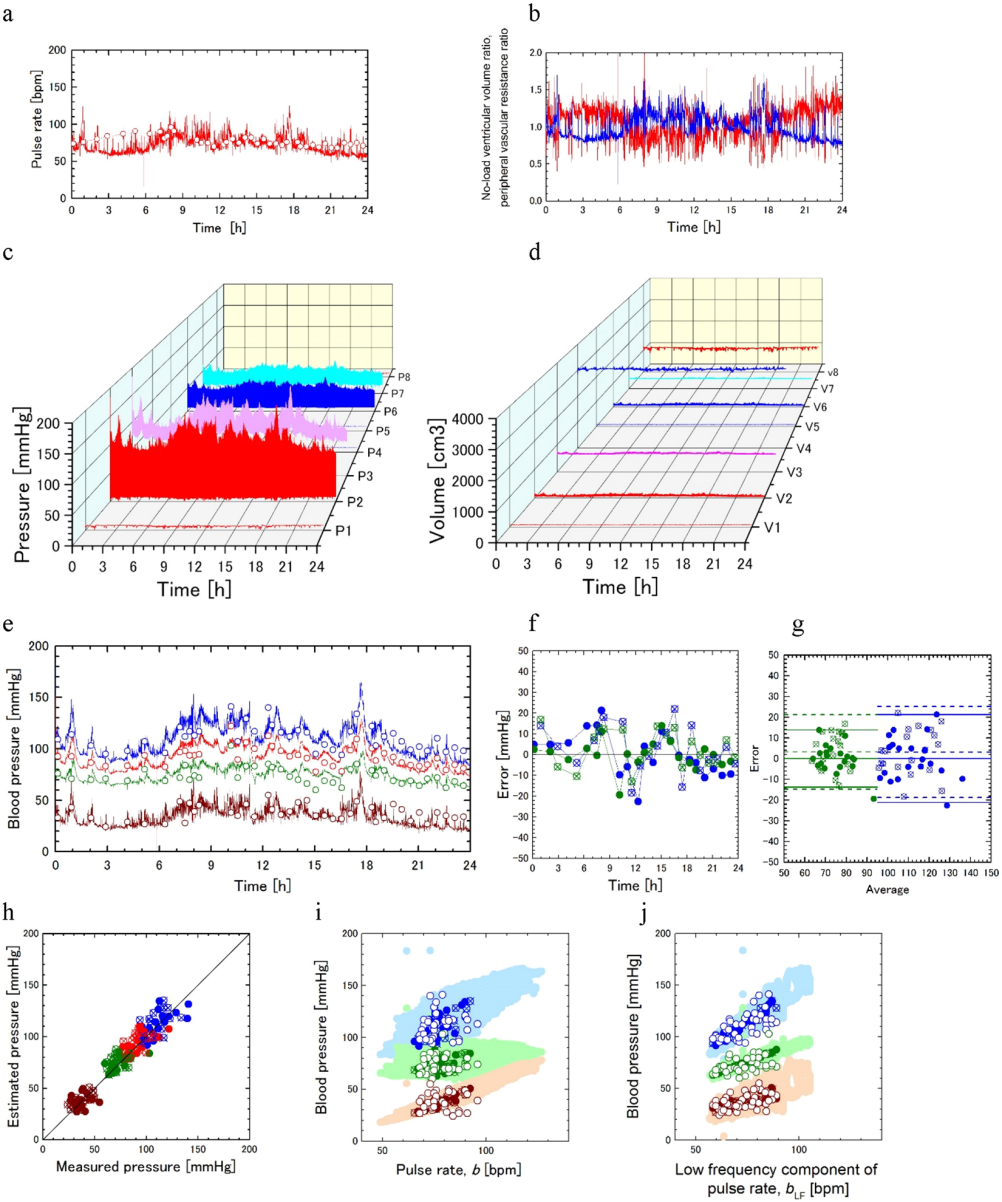
Daily continuous blood pressure (DCBP) estimation for subject 1 in day 1. **(a)** Pulse rate measurements of wearable device (line) and those of sphygmomanometer (circles). (**b**) Computational results for no-load ventricular volume ratio (blue) and peripheral vascular resistance ratio (red). Pressures (**c**) and volumes (**d**) of eight elastic containers. (**e)** 24 hour computations (lines) and measurements by sphygmomanometer (circles) for systolic (blue), average (red), diastolic (green), and pulse (brown) pressures. Same colors are used in (**f**-**j**). (**f**) Variation of estimation errors of computations for systolic and diastolic pressures at time points for parameter determination (closed circles) and those for validation (cross circles). (**g**) Bland-Altman plot showing the relation between errors and averages of estimations and measurements for systolic and diastolic pressures with mean values (middle lines) and mean values ±2 × standard deviation (upper and lower lines) with data for parameter determination data (closed circles, solid lines) and validation data (cross circles, broken lines). (**h**) Correlation between measurements and calculations for systolic, mean, diastolic, and pulse pressures using the same symbols as those in (**g**). Measurements (open circles), corresponding computations (closed circles), and all 24 hour computations (light color circles) plotted with pulse rate (**i**) and with low frequency component of pulse rate (**j**).

We first show the results of the subject 1. Variation of the pulse rate in 24 hours of the day 1 is shown in Fig. 2(a). Approximate times of activities in the day are, sleeping in 0:00–7:30, wake up in 7:30–24:00, meals at 8:00, 12:00, and 18:30, driving car at 9:00 and 18:00, office work in 10:00–17:30. Measurements of the wearable device (line) and those of the sphygmomanometer (circles) agree well. Differential equations of the present model were integrated using the measurement data of the pulse rate by the wearable device. Values of the model parameters, ${b}_{0}$, ${E}_{30}$, ${R}_{40}$, ${s}_{a}$, ${s}_{r}$, and ${T}_{c}$, were determined by comparing the result of the sphygmomanometer measurements and those of the computations performed with various combinations of these parameters. Variations of the no-load ventricular volume ratio $a(t)$ and the peripheral vascular resistance ratio $r(t)$ are shown in Fig. 2b. Computational results for the variations of pressures ${P}_{i}(t)$ and volumes ${V}_{i}(t)$ of eight elastic containers in the model are shown in Fig. 2c,d, respectively. Lines in Fig. 2e show computational results of the daily continuous blood pressure (DCBP) estimation for variation of the systolic (blue), average (red), diastolic (green), and pulse (brown) pressures at all pulses in 24 hours obtained from the result of the arterial pressure ${P}_{3}(t)$ (Fig. 2c). Circles in the figure are corresponding measurement data by the sphygmomanometer.

In order to obtain the blood pressure estimation at a fixed time point, computation is necessary using pulse rate data in a certain period. According to preliminary calculations, it was confirmed that the computational result in the last 60 s of 120 s calculation starting from the time point 60 s ahead of the target time point using the initial value of the low frequency component of the pulse rate variation evaluated by the data in the period of 1,600 s, which is eight times of the time constant ${T}_{c}$, agreed well with the corresponding results of the 24 hours calculation. The average values in the last 60 s were used in the comparison with the measurement data of sphygmomanometer with one minute temporal resolution.

**Figure 3:**
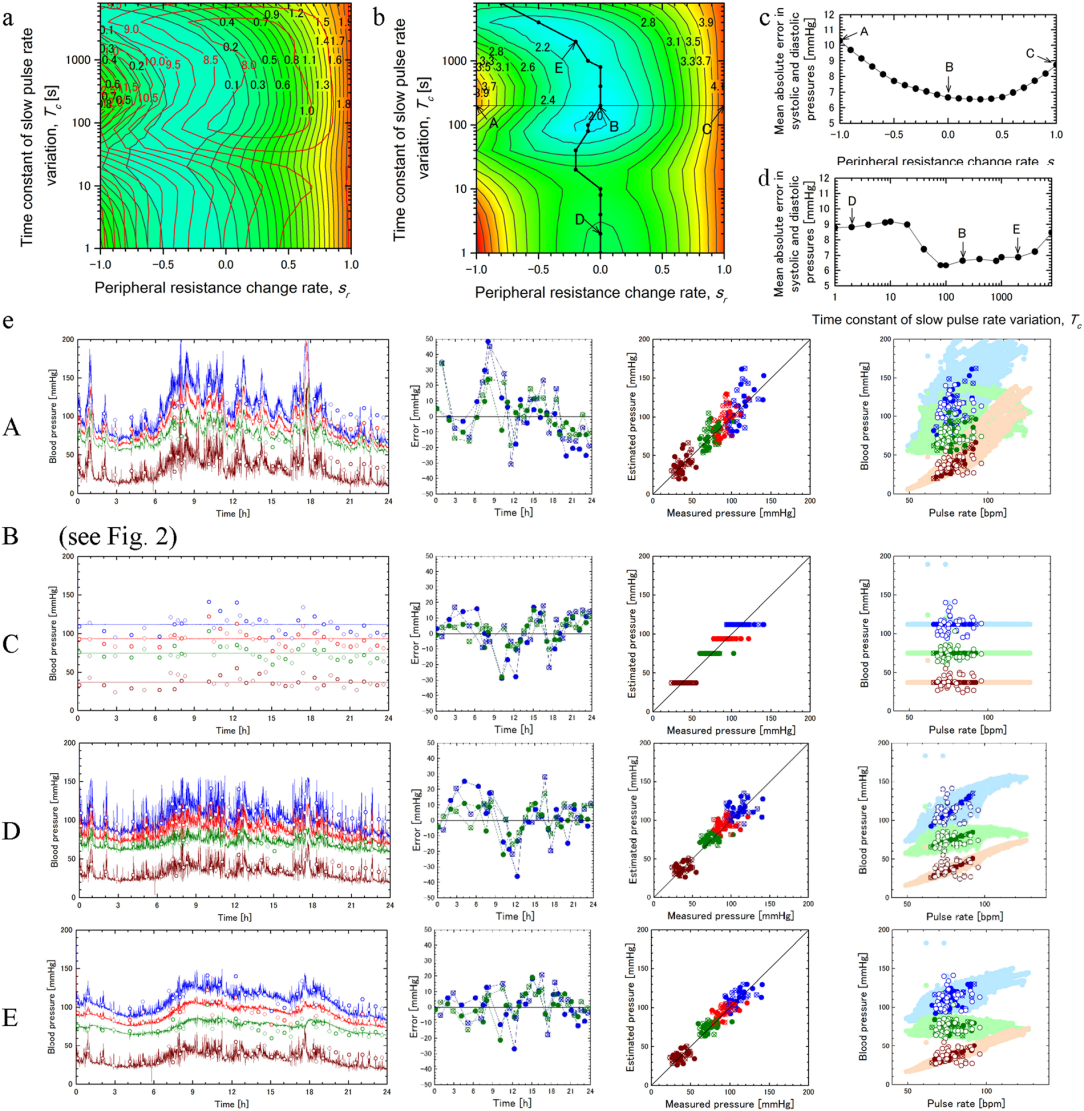
Effects of model parameters on DCBP estimation for subject 1 in day 1. Distributions of cost functions ${J}_{sd}$ (black lines) and ${J}_{rms}$ (red lines) (**a**) and those of ${J}_{sum}$ (**b**) with peripheral resistance change rate ${s}_{r}$ and slow pulse rate variation time constant ${T}_{c}$. Labels A, B, C, D, E in (**b)** correspond to those in (**c-e)**. The polyline in **b** connects the points with ${s}_{r}$ which minimizes the cost function ${J}_{sum}$ for each value of ${T}_{c}$. (**c)** Variation of mean absolute error of the computation for systolic and diastolic pressures as a function of ${s}_{r}$ for fixed ${T}_{c}=200\,[s]$, and (**d)** that of ${T}_{c}$ along the polyline in (**b). (e)** Comparison between results in cases A-E for 24 hour estimations and measurements of the systolic (blue), average (red), diastolic (green), and pulse (brown) pressures (fist column), errors in the calculated systolic and diastolic pressures (second column), correlation between the measured and calculated results (third column), and the correlations of the measured and calculated blood pressures with the pulse rate (fourth column). Refer to the legend of Fig. 2 for details.

Errors of the computations from measurements of the sphygmomanometer at 40 time points are shown in Fig. 3f for the systolic and diastolic pressures. Colors of the plots correspond to those in Fig. 2e, and closed and cross circles show the data used for parameter determination (20 time points) and those for validation (20 time points), respectively. Figure 2g (Bland-Altman plot) shows the relation between errors and averages for estimation and measurement for systolic (blue) and diastolic (green) pressures with mean values (middle lines) and mean values ±2 × standard deviation (upper and lower lines) with data for parameter determination (closed circles, solid lines) and those for validation (cross circles, broken lines). Rows 1–4 in Table 1 show mean values and standard deviations of measurements, estimations, and estimation errors for systolic and diastolic pressures in parameter determination data and those for validation ones.

**Table 1:** DCBP estimation for subject 1 in day 1.

		Param. determ. data			Validation data		
		Meas.	Estim.	Error	Meas.	Estim.	Error
Systolic	MEAN	112.0	112.6	0.0	109.4	112.0	3.2
	SD	13.3	12.6	10.6	10.9	11.8	11.0
Diastolic	MEAN	74.9	75.1	0.0	71.9	74.9	3.3
	SD	9.7	7.5	6.9	6.5	6.7	9.0
Independent variables	Dependent variables	Param. determ. data			Validation data		
		*r*	*R*^2^	slope	*r*	*R*^2^	slope
Meas. data	*P*_sys_	0.646	0.417	0.576	0.570	0.325	0.660
	*P*_dia_	0.702	0.492	0.485	0.177	0.031	0.205
	*P*_ave_	0.713	0.509	0.588	0.440	0.194	0.551
	*P*_pulse_	0.114	0.013	0.097	0.705	0.496	0.505
Independent variables	Dependent variables	Estimations			Measurements		
		*r*	*R*^2^	slope	*r*	*R*^2^	slope
Pulse rate	*P*_sys_	0.635	0.403	1.173	0.083	0.007	0.145
	*P*_dia_	0.361	0.130	0.388	0.069	0.005	0.083
	*P*_pulse_	0.878	0.771	0.785	0.057	0.003	0.062
Low frequency component of pulse rate	*P*_sys_	0.969	0.940	1.307	0.606	0.368	0.817
	*P*_dia_	0.997	0.994	0.782	0.454	0.206	0.420
	*P*_pulse_	0.805	0.648	0.525	0.471	0.222	0.397

Rows 1–4 show mean values and standard deviations of measurements, estimations, and estimation errors for systolic and diastolic pressures for parameter determination data and those for validation ones. Following rows show correlations among measured and estimated blood pressures and pulse rate with Pearson’s correlation coefficient *r*, coefficient of determination *R*^2^, and slope of regression line. Rows 5–8 correspond to the results in Fig. 2h for parameter determination data and those for validation ones. Rows 9–11 and 12–14 correspond to the results for computations and measurements in Fig. 2i,j, respectively.

Correlations between estimations and measurements for systolic (blue), mean (red), diastolic (green), and pulse (brown) pressures are shown in Fig. 2h using the same symbols as those in Fig. 2g. Figure 2i,j show the measurements (open circles), corresponding calculations (closed and cross circles), and all the 24 hour calculations (light color circles) for the systolic (blue), diastolic (green), and pulse (brown) pressures plotted with the pulse rate and with the low frequency component of the pulse rate variation, respectively. Rows 5–14 in Table 1 show correlations among measured and estimated blood pressures and pulse rate with Pearson’s correlation coefficient *r*, coefficient of determination *R*^2^, and slope of regression line. Rows 5–8 correspond to the results in Fig. 2h for parameter determination data and those for validation data. Rows 9–11 and 12–14 correspond to the results for computations and measurements in Fig. 2i,j, respectively.

### Effect of model parameters on DCBP estimation for subject 1 in day 1

In the above-mentioned results for subject 1 in day 1, the set of model parameters, ${b}_{0}$, ${E}_{30}$, ${R}_{40}$, ${s}_{a}$, ${s}_{r}$, and ${T}_{c}$, which satisfy the conditions (1)–(5) mentioned in method section best were determined by comparing the result of the sphygmomanometer measurement and those of the computations performed with the pulse rate data by the wearable device and various combinations of these parameters (Fig. 3). Computation was performed by changing the peripheral resistance change rate ${s}_{r}$ from −1 to 1 with the interval of 0.1 and the slow pulse rate variation time constant ${T}_{c}\,[{\rm{s}}]$ from 1 to 8,000 as 1, 2, 4, 8, 10, 20, 40, 80, 100, 200, 400, 800, 1,000, 2,000, 4,000, 8,000 in 21 × 16 = 336 cases. According to the preliminary computations, the stroke volume change rate ${s}_{a}$ is given by the empirical expression.

1 $${s}_{a}=1-{s}_{r}$$

In determination of the model parameters, ${E}_{30}$ and ${R}_{40}$ were first determined to satisfy condition (2) to minimize errors in mean values of the average and pulse pressures for all cases. As to condition (3) to minimize errors in standard deviations and mean square errors of the average and pulse pressures, distributions of the cost functions ${J}_{sd}$ (black lines) and ${J}_{rms}$ (red lines) of the peripheral resistance change rate ${s}_{r}$ and the slow pulse rate variation time constant ${T}_{c}$ were shown in Fig. 3a.

2 $$\begin{array}{c}{J}_{sd}={({\sigma }_{pulsec}/{\sigma }_{pulsem}-1)}^{2}+{({\sigma }_{avec}/{\sigma }_{avem}-1)}^{2}\\ {J}_{rms}=\frac{1}{2N}\sum \left\{{({P}_{pulsem}({t}_{n})-{P}_{pulsec}({t}_{n}))}^{2}+{({P}_{avem}({t}_{n})-{P}_{avec}({t}_{n}))}^{2}\right\}\end{array}$$

The former function evaluates the degree of agreement for the standard deviations ${\sigma }_{pulsec}$ and ${\sigma }_{avec}$ of ${P}_{pulsec}({t}_{n})$ and ${P}_{avec}({t}_{n})$ with those of measurement ${\sigma }_{pulsem}$ and ${\sigma }_{avem}$, respectively, and the latter function evaluates the mean square errors of ${P}_{pulsec}({t}_{n})$ and ${P}_{avec}({t}_{n})$. Furthermore, distribution of the cost function ${J}_{sum}$ defined as the weighted sum of these functions are shown in Fig. 3b.

3 $${J}_{sum}={J}_{sd}+\alpha {J}_{rms}$$

The weighting factor ${\rm{\alpha }}$ was determined as 0.25 [Pa^−2^] by preliminary calculations. The polyline including B, D, and E in Fig. 3b connects the points with the value of ${s}_{r}$ which minimizes the cost function ${J}_{sum}$ for each value of ${T}_{c}$.

Figure 3c shows the variation of the mean absolute error of the computations for systolic and diastolic pressures with the peripheral resistance change rate ${s}_{r}$ for the time constant ${T}_{c}=200\,[s]$ corresponding to the line A-C in Fig. 3b. Figure 3d shows the variation of the mean absolute error of the computations for systolic and diastolic pressures along the polyline in Fig. 3b as a function of the time constant.

Figure 3e compares the cases A, B, C, D, and E for the 24 hour computational results of the systolic (blue), average (red), diastolic (green), and pulse (brown) pressures (first column), the errors in the estimated systolic and diastolic pressures (second column), the correlation between estimated blood pressures and measured ones (third column), and the correlations of measured blood pressures and estimated ones with the pulse rate (fourth column). Refer to Fig. 2 for the results of case B.

### Verification by measurement data for subject 1 in four days

**Figure 4:**
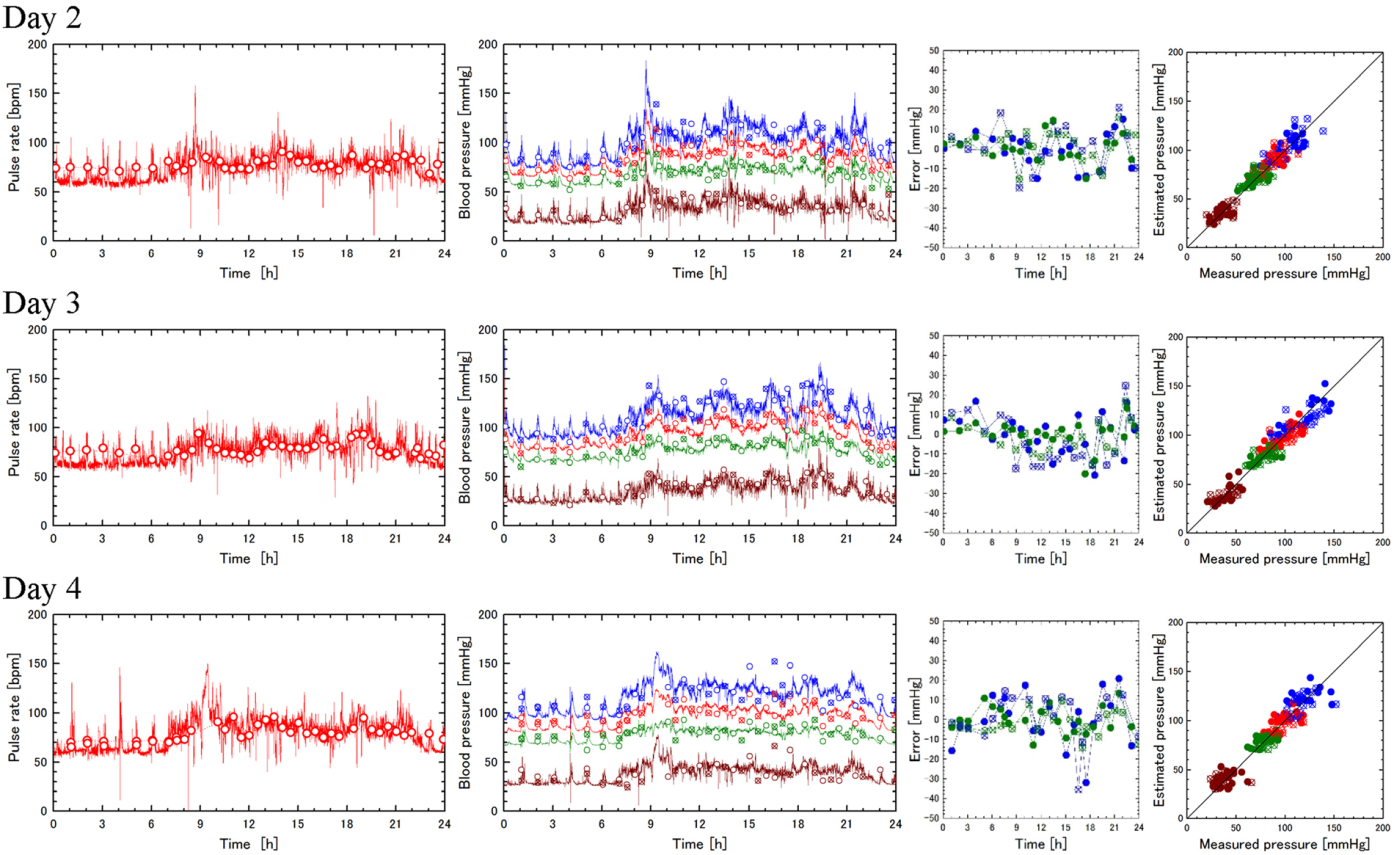
DCBP estimation for subject 1 in four days. Comparison between days 2–4 for pulse rate measurements (first column) by wearable device (lines) and sphygmomanometer (circles), blood pressure variations (second column) by computations (lines) and sphygmomanometer measurements (circles), errors in calculated systolic and diastolic blood pressures (third column), and correlations between measured and calculated blood pressures (fourth column). Refer to the legend of Fig. 2 for details.

Determination of the model parameters of the subject 1 were performed for the experiments in day 2 – day 4 in the same way as that of day 1. Daily activities and their approximate times in these days are almost the same as those in day 1. Measurement of the pulse rate by the wearable device and the sphygmomanometer, the 24 hour computation and measurement of the blood pressures, the errors in the estimated blood pressures, and the correlation between the measured and estimated blood pressures are shown in Fig. 4. Table 2 compares the six model parameters, ${b}_{0}$, ${E}_{30}$, ${R}_{40}$, ${s}_{a}$, ${s}_{r}$, and ${T}_{c}$; and two associated parameters ${P}_{sys0}$ and ${P}_{dia0}$ (cf. Eq. (20) in method section) (rows 1–8), mean values (MEAN), standard deviations (SD), and mean absolute values (MA) of estimation errors for systolic and diastolic pressures obtained for validation data with daily determined model parameters (rows 9–14) and those with mean parameters (rows 15–20).

**Table 2:** DCBP estimation for subject 1 in four days.

		Day 1	Day 2	Day 3	Day 4	MEAN	SD
*b*_0_ [bpm]		73	74	76	79	75.5	2.6
*E*_30_ [Pa/cm^3^]		94	86	98	109	96.8	9.6
*R*_40_ [Pa·s/cm^3^]		151	132	150	150	145.8	9.2
*s*_a_ [-]		1.0	1.2	1.0	0.7	1.0	0.2
*s*_r_ [-]		0	−0.2	0	0.3	0.0	0.2
*T*_*c*_ [s]		200	200	200	200	200.0	0.0
*P*_sys0_ [mmHg]		109	99	113	118	109.8	8.1
*P*_dia0_ [mmHg]		75	67	77	79	74.5	5.3
**Estimation errors with daily model parameters [mmHg]**							
Systolic	MEAN	3.2	1.9	−3.0	1.1	0.8	2.7
	SD	11.1	10.2	12.2	11.4	11.2	0.8
	MA	8.9	7.9	10.7	8.4	9.0	1.2
Diastolic	MEAN	3.3	2.2	−1.8	−2.3	0.4	2.8
	SD	9.0	7.9	7.6	6.0	7.6	1.2
	MA	7.9	6.2	6.1	4.6	6.2	1.3
**Estimation errors with mean model parameters [mmHg]**							
Systolic	MEAN	−1.1	7.7	−5.4	−0.9	0.1	5.5
	SD	10.7	9.9	12.3	13.3	11.6	1.5
	MA	8.6	7.2	10.8	11.1	9.4	1.9
Diastolic	MEAN	0.8	7.0	−3.8	−3.8	0.1	5.1
	SD	8.8	7.6	7.7	7.0	7.8	0.8
	MA	7.7	6.1	6.1	5.7	6.4	0.9

Comparison of six model parameters and two associated parameters (rows 1–8), mean values (MEAN), standard deviations (SD), and mean absolute values (MA) of estimation errors for systolic and diastolic pressures obtained for validation data with daily determined model parameters (rows 9–14) and those with mean parameters (rows 15–20).

### Verification by measurement data for five subjects in one day

**Figure 5:**
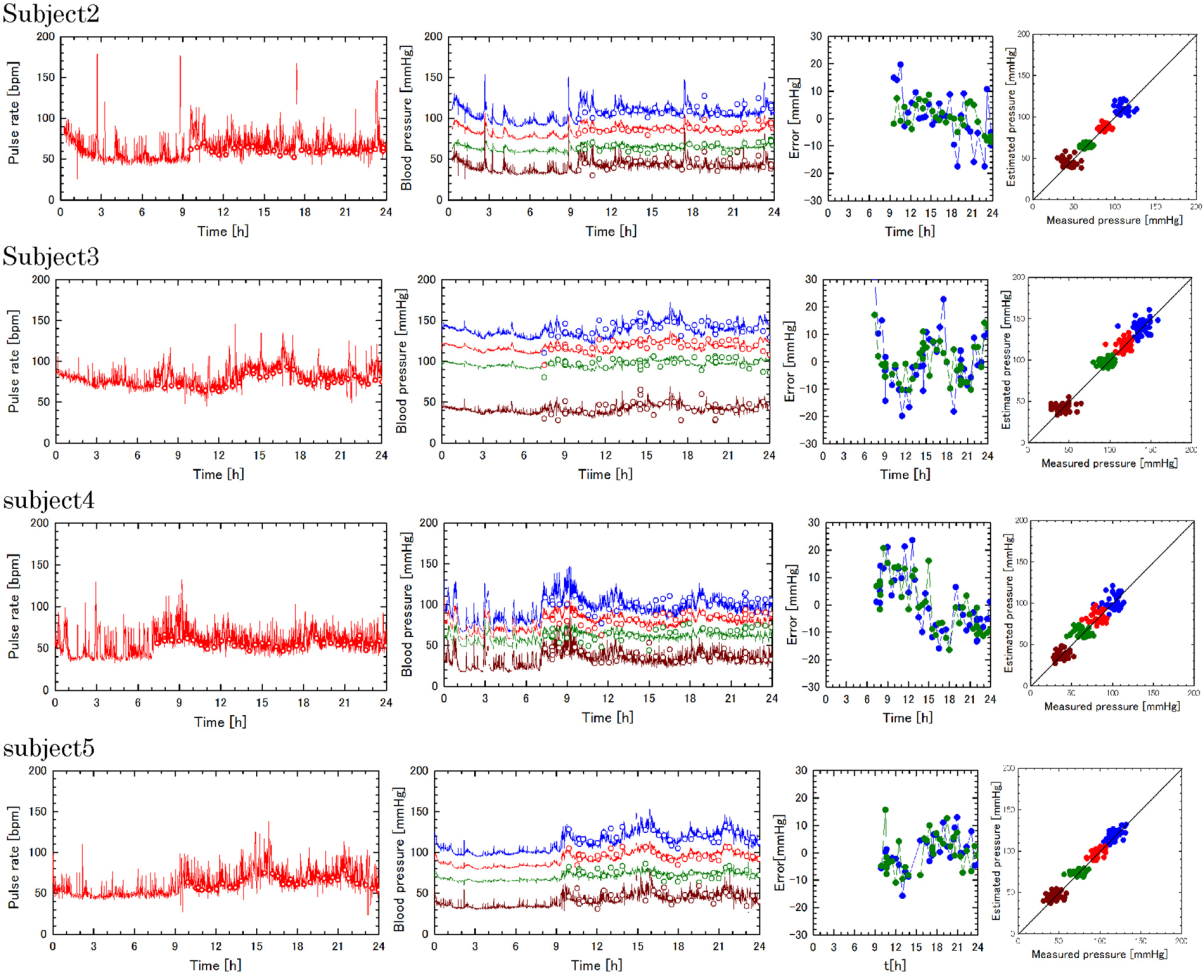
DCBP estimations for four subjects in one day. Refer to the legend of Fig. 4 for details.

Another experiment was performed for three male volunteers of 20 s and one male volunteer of 40 s (subjects 2–5) in the same way excepting that sphygmomanometer measurement was performed during only wake up hours in one day. Wake up hours were recorded for the daily activity for subjects 2–5. Determination of the model parameters of the subjects 1–5 were performed for the experiments in the same way as the former ones. Measurement of the pulse rate by the wearable device and the sphygmomanometer, the 24 hour computation and measurement of the blood pressures, the errors in the estimated blood pressures, and the correlation between the measured and estimated blood pressures are shown in Fig. 5. Comparison of six model parameters and two associated parameters (rows 1–8), mean values (MEAN), standard deviations (SD), and mean absolute values (MA) of estimation errors for systolic and diastolic pressures (rows 9–14) are shown in Table 3.

**Table 3:** DCBP estimation for five subjects in one day.

	Subject 1	Subject 2	Subject 3	Subject 4	Subject 5	MEAN	SD
*b*_0_ [bpm]	73	60	75	56	62	65.2	8.3
*E*_30_ [Pa/cm^3^]	98	108	111	87	115	103.8	11.3
*R*_40_ [Pa·s/cm^3^]	149	158	192	157	175	166.2	17.3
*s*_a_ [-]	0.9	0.5	0.5	0.8	0.5	0.6	0.2
*s*_r_ [-]	0.1	0.5	0.5	0.2	0.5	0.4	0.2
*T*_*c*_ [s]	200	200	200	200	200	200.0	0.0
*P*_sys0_ [mmHg]	109	101	135	92	112	109.8	16.1
*P*_dia0_ [mmHg]	73	62	97	59	71	72.4	15.0
**Estimation errors [mmHg]**							
Systolic MEAN	0.0	0.0	0.0	0.0	0.0	0.0	0.0
SD	10.8	9.2	10.3	10.4	5.8	9.3	2.0
MA	8.8	7.0	7.7	8.6	4.4	7.3	1.8
Diastolic MEAN	0.0	0.0	0.0	0.0	0.0	0.0	0.0
SD	7.9	9.2	7.0	9.6	6.3	8.0	1.4
MA	6.3	4.2	5.7	8.0	5.1	5.9	1.4

Comparison of six model parameters and two associated parameters (rows 1–8), mean values (MEAN), standard deviations (SD), and mean absolute values (MA) of estimation errors for systolic and diastolic pressures obtained with measurement data in wake up hours for each subject (rows 9–14).

## Discussion

In this study we proposed a novel method to obtain daily continuous blood pressure (DCBP) based on a simple circulatory system model using pulse rate measurement in order to overcome the limitations of existing methodologies, and showed that it provides appropriate estimation of DCBP. The present model consists of a circulatory dynamic system model and an inverse model of a circulatory control system with input of pulse rate and six model parameters. Computation was performed with the model using 24-hour pulse rate measurements for one subject in four days and using those for four subjects in one day to obtain the optimum parameters and then to verify the proposed method in comparison with sphygmomanometer measurements.

Evidence of the validity of the present model is that computational results for systolic, average, diastolic, and pulse pressures for subject 1 in day 1 agree with those of measurements with a sphygmomanometer as shown in Fig. 2e,h. The computational results for daily variation of systolic pressure show reduced blood pressure in the sleeping hours^3^ and fluctuation of the blood pressure in the wake up hours^27^ (Fig. 2e). Computational results for the variations of pressures ${P}_{i}(t)$ and volumes ${V}_{i}(t)$ of eight elastic containers in the model are qualitatively in good agreement with those of the literature^28,29^ (Fig. 2c,d). Variation of the no-load ventricular volume ratio $a(t)$ and that of the peripheral vascular resistance ratio $r(t)$ show increase of the no-load ventricular volume and decrease in the peripheral vascular resistance in the systemic and pulmonary arteries with increasing pulse rate through the circulatory control inverse model, respectively (Fig. 2b).

As shown in Table 1, the standard deviation of measurements for the systolic pressure with parameter determination data and that with validation data were 13.3 mmHg and 10.9 mmHg, respectively, and those for the diastolic pressure were 9.7 mmHg and 6.5 mm Hg, respectively. Corresponding values for the errors of computations from measurements were 10.6 mmHg and 11.0 mmHg for the systolic pressure, and those for diastolic pressure were 6.9 mmHg and 9.0 mmHg, respectively, which are comparable with those of measurements. These standard deviations of the errors are less than 11.3 mmHg, which is evaluated assuming the standard deviation of a common sphygmomanometer (8 mmHg) for those of measurements and computations, and independency between them.

4 $${\sigma }_{cal-meas}=\sqrt{{\sigma }_{cal}^{2}+{\sigma }_{meas}^{2}}=\sqrt{{8}^{2}+{8}^{2}}=11.3\,({\rm{mmHg}})$$

Mean value for the errors evaluated with validation data was 3.2 mmHg for the systolic pressure, and that for diastolic pressure was 3.3 mmHg, respectively, which are less than the tolerance of common sphygmomanometers (5 mmHg).

The correlation coefficients and the coefficients of determination have large values to show the effectiveness of the present estimation method as shown in Fig. 2h and rows 5–8 of Table 1. The correlation between the pulse rate and the blood pressure has been considered to be low^19^. As to the measurements and corresponding calculations, correlations with the pulse rate are low as shown in Fig. 2i and rows 9–11 of Table 1, being consistent with former studies. On the other hand, they have significant correlations with the low frequency component of the pulse rate variation in Fig. 2j and rows 12–14 of Table 1, in accordance with the circadian cycle of the pulse rate^30^ and that of the blood pressure^31^. As to all the 24 hour calculations, the systolic and diastolic pressures have more significant correlations with the low frequency component of the pulse rate whereas the pulse pressure has the one with the pulse rate, reflecting the present circulatory control inverse model.

Effect of model parameters on DCBP estimation is discussed below. We first investigate the effect of the peripheral resistance change rate ${s}_{r}$ with the time constant ${T}_{c}$ fixed to 200 s. The cost function ${J}_{sum}$ takes the minimum value at B with ${s}_{r}=0$ as shown in Fig. 3b in the range between A and C. The mean absolute error of the estimation for the systolic and diastolic pressures takes the minimum at somewhat larger value of ${s}_{r}=0.3$ in the same range as shown in Fig. 3c. As shown in the first two figures in the result of case A with ${s}_{r}=-\,1$ and ${s}_{a}=2$ (cf. Equation (1)) in Fig. 3e, fluctuations of the calculated results for the systolic, average, diastolic, and pulse pressures are larger than those of the measurement since the systemic peripheral resistance and the stroke volume substantially increase with increasing pulse rate. On the other hand, in the result of case C with ${s}_{r}=1$ and ${s}_{a}=0$, the calculated results show zero fluctuations which are smaller than those of the measurement as the systemic peripheral resistance changes inversely proportional to the pulse rate and the stroke volume is constant with the change of pulse rate. The result of case B with ${s}_{r}=0$ and ${s}_{a}=1$ has the characteristics between those of A and C, showing good agreement with that of the measurement as shown in Fig. 2. Next we investigate the effect of the time constant ${T}_{c}$. As shown in Fig. 3d, the error takes relatively high values in the lower range of the time constant below 20 s, then reduces in the middle range up to 80 s, and remains low in the higher range in the figure. It is, therefore, reasonable to consider that the appropriate value of the time constant is larger than 80 s. The result of case D with ${T}_{c}=2\,[{\rm{s}}]$ in Fig. 3e shows large fluctuations in the 24 hour computation in the first column and very significant correlations with the pulse rate in the last column, both of which do not agree with those of measurement. In the result of case E with ${T}_{c}=2,000\,[{\rm{s}}]$ in Fig. 3e, the 24 hour variation of the blood pressure in the first column appears too smooth to properly represent the fluctuations in the measurement result. Therefore, the appropriate value of the time constant is considered to be smaller than 2,000 s. According to the above-mentioned discussion, we expediently determined the value of the time constant ${T}_{c}$ as 200 [s] corresponding to the case B. Since this parameter in the higher range is not very sensitive to the result of the blood pressure estimation, the same value was used for the analyses of day 2–4 of subject 1 and those of subjects 2–5.

As to comparison among DCBP estimations for subject 1 in four days, similar results were obtained for pulse rate measurements, systolic, average, diastolic, and pulse pressure estimations, errors in calculated systolic and diastolic blood pressures, and correlations between measured and calculated blood pressures as shown in Fig. 4. The model parameters fluctuate in four days as shown in Table 2, reflecting daily variation of the personal conditions of the blood vessel elasticity and peripheral resistance in the circulatory dynamics system and those of the pulsation and resistance control in the circulatory control system. Therefore, the average values and deviations of these parameters during sufficiently long period for a specific person can be used as reliable personal vital signs each of which has a clear physiological meaning relating DCBP. We expect that these parameters collected for a large number of subjects can be used for fundamental studies such as the relation between the parameters and some disease, and for clinical applications such as diagnosis based on these studies. Mean value and standard deviation of the errors evaluated with validation data averaged in four days are 0.1 mmHg and 11.6 mmHg for the systolic pressure and 0.1 mmHg and 7.8 mmHg for the diastolic pressure, respectively (Table 2). These standard deviations are almost equal to or less than the above-mentioned value of 11.3 mmHg (Eq. (4)) evaluated assuming the standard deviation of common sphygmomanometers for those of measurements and computations, and independency between them. The mean values are also less than the tolerance of common sphygmomanometers (5 mmHg). Mean absolute error of computations evaluated with validation data averaged in four days are 9.4 mmHg for systolic pressure and 6.4 mmHg for diastolic pressure, respectively (Table 2). Although the present verification is very limited for one subject using sphygmomanometer data for the standard, these values are comparable with that of the standard for wearable, cuffless blood pressure measuring devices of 7 mmHg^32^.

As to comparison among DCBP estimations for five subjects in one day, similar results were obtained for pulse rate measurements, systolic, average, diastolic, and pulse pressure estimations, errors in calculated systolic and diastolic blood pressures, and correlations between measured and calculated blood pressures as shown in Fig. 5. The model parameters deviate among five subjects as shown in Table 3 reflecting the blood vessel elasticity and peripheral resistance in the circulatory dynamics system and those of the circulatory control system for each subject. Mean value, standard deviation, and mean absolute value of the estimation errors evaluated with daytime measurement data and averaged in five subjects are 0.0 mmHg, 9.3 mmHg, and 7.3 mm Hg for systolic pressure and 0.0 mmHg, 8.0 mmHg, and 5.9 mmHg for diastolic pressure, respectively (Table 3). The mean values are null since the same measurement data was used for parameter determination and validation in this preliminary experiment for five subjects. The standard deviations are less than the above-mentioned value of 11.3 mmHg (Eq. (4)). Although the present verification is very limited for five subject using the same sphygmomanometer data in daytime for the standard of data determination and validation, the mean absolute values are comparable with that of the standard for wearable, cuffless blood pressure measuring devices 7 mmHg^32^.

Our results suggest that a fundamental part of DCBP can be represented by continuous pulse rate data and the simple circulatory dynamics and circulatory control inverse model with six model parameters. It is obviously easier to perform DCBP estimation by this method than by the other methods.

As limitations of the present work, the result of model parameter determination is influenced by the constraint between model parameters ${s}_{a}$ and ${s}_{r}$ (Eq. (1)), the definition of the evaluation function ${J}_{sum}$ (Eq. (3)) and the value of the weight ${\rm{\alpha }}$, and the assumption of uniformity of the time constant ${T}_{c}$. Errors in measurement of systolic and diastolic pressures and pulse rate with the automatic sphygmomanometer in sitting position influence the result of model parameter determination and that of validation. Errors in measurement of pulse rate with the wearable device influence the results of blood pressure estimation and parameter determination, and those of validation. In this study we proposed a DCBP estimation method based on pulse rate measurement and verified it by a preliminary experiment for one subject in four days and that for additional four subjects in wake up hours in one day. These experiments are insufficient for further statistical analysis. The present method to determine personal model parameters is not suitable for real usage in DCBP estimation devices since the method requires sphygmomanometer measurements over 24 hours or wake up hours.

As a future work, confirmation experiment for a sufficient number of subjects is necessary to verify the validity of the method to determine model parameters and to clarify the accuracy of the present blood pressure estimation method, statistical characteristics of the model parameters and those of their temporal variations. Other confirmation experiments using a mercury sphygmomanometer and a Holter electrocardiograph are also necessary to evaluate the effect of the accuracy of measurement for the standard blood pressure or that for the pulse rate on the analysis results, respectively. For application to DCBP estimation devices, study is necessary for the model parameters determination method such as a successive parameter update procedure instead of the present batch one.

## Conclusions

In conclusion, we constructed the simple model of the circulatory dynamic system and the circulatory control inverse system with input of the pulse rate for the purpose of DCBP estimation. Validity of the DCBP estimation method was examined by preliminary experiment. The input of the model or the continuous pulse rate variation was measured by the wearable device for one subject in four days and four subjects in one day. Systolic and diastolic pressures and pulse rate were also measured with the sphygmomanometer with the interval of 30 or 60 min. Differential equations of the model were integrated using the measured pulse rate data. Values of six model parameters were determined for each of data by comparing the result of the sphygmomanometer measurement and those of the computations for various combinations of these parameters. Although the present verification is very limited, the mean absolute error was comparable with that of the standard for wearable, cuffless blood pressure measuring devices^32^. Our results demonstrate how DCBP is appropriately estimated by the simple circulatory system model and the pulse rate measurement. We anticipate our methodology to be a starting point of new diagnosis based on DCBP^3,4,33,34^. Studies to clarify the relation between DCBP and diseases are important in many clinical departments. Furthermore, present six model parameters can be used as reliable personal vital signs relating the blood pressure, measurement of which often experiences large fluctuations^5^.

## Methods

### Circulatory dynamics model

The circulatory dynamics model consists of eight elastic containers representing a left atrial (1), a left ventricle (2), systemic arteries (3), systemic veins including organs (4), a right atrial (5), a right ventricle (6), pulmonary arteries (7), and pulmonary veins (8); and eight liner resistors connecting these containers. The numbers of resistors are identical to those of downstream side containers.

Dynamics of the pressures in containers are represented by the following equations.

5 $$\frac{d{P}_{i}}{dt}={E}_{i}\left({Q}_{i}-{Q}_{i+1}-\frac{d{V}_{i}}{dt}\right)\,(i=1,\cdots ,8).$$

In the above equation ${V}_{i}(t)\,(i=2,\,6)$ represent the variations of the volume at zero pressure, or no-load volume, of the left and right ventricles, respectively, given by

6 $${V}_{i}(t)={f}_{i}(0)+a(t)\left\{{f}_{i}\left(\frac{b(t)\tau (t)}{{b}_{0}{\tau }_{0}}\right)-{f}_{i}(0)\right\}\,(i=2,6)\,,$$

where $b(t)$ is the pulse rate of the pulse including the time point $t,\,\tau (t)$ is the elapsed time from the beginning of this pulse, ${b}_{0}$ and ${\tau }_{0}$ are the standard pulse rate and the corresponding cardiac cycle, respectively, ${f}_{i}(\tau (t)/{\tau }_{0})$ are the variations of the no-load volume of the left and right ventricles for the standard pulse rate derived by reference to the literature^23^ (Fig. 1b), $a(t)$, we call it as *no-load stroke volume ratio*, is the ratio of the ventricular volume change to that for the standard pulse rate, determination of the value of which by the circulatory control inverse model will be explained later. No-load volumes of the other containers are constant.

7 $${V}_{i}(t)={V}_{i0}\,(i=1,3,5,7,8)$$

In Eq. (5), ${E}_{i}\,(i=1\cdots 8)$ are the elasticities of the containers. The elasticity of the left ventricle ${E}_{2}$ is assumed to take a relatively lower value for the internal pressure lower than a threshold due to buckling.

8 $${E}_{2}=\left\{\begin{array}{ll}{E}_{2low} & {P}_{2}\le {P}_{20}\\ {E}_{2high} & {P}_{2} > {P}_{20}\end{array}\right.$$

The elasticity of lumped systemic arteries ${E}_{3}$ is defined as a function of the arterial pressure ${P}_{3}$.

9 $${E}_{3}=\left\{\begin{array}{ll}{E}_{30}{P}_{3}/{P}_{3aves} & {P}_{3}\ge {P}_{3aves}/2\\ \frac{{E}_{30}({P}_{3aves}-{P}_{3})}{{P}_{3aves}} & {P}_{3} < {P}_{3aves}/2\end{array}\right.$$

where the first expression corresponds to the real characteristics of the arteries in the real range of the pressure^35^ whereas the second one is given expediently to prevent divergence of the calculation.

Elasticities of the other containers are assumed to be constant for simplicity.

10 $${E}_{i}={E}_{i0}\,(i=1,4,5,6,7,8)$$

The flow rates ${Q}_{i}$ through the resistors are given as

11 $${Q}_{i}=\frac{{C}_{i}({P}_{i-1}-{P}_{i})}{{R}_{i}}\,(i=1\cdots 8)$$

where ${C}_{i}$ represent check valve characteristics to prevent reverse flow.

12 $${C}_{i}=\left\{\begin{array}{ll}0 & {P}_{i-1}\le {P}_{i}\\ 1 & {P}_{i-1} > {P}_{i}\end{array}\right.$$

Indices $i=2,\,3,\,6,\,7$ correspond to the mitral, aortic, tricuspid, and pulmonary valves, respectively. In the other resistors there is no possibility of reverse flow.

${R}_{i}\,(i=1\cdots 8)$ are the resistance coefficients. Those of the peripheral systemic (4) and pulmonary (8) arteries, respectively, are modeled by the following expression.

13 $${R}_{i}={R}_{i0}r(t)/a(t)\,(i=4,8)$$

where $r(t)$, we call it as *peripheral vascular resistance ratio*, is the ratio of the peripheral vascular resistance coefficient to that for the standard pulse rate multiplied with the no-load stroke volume ratio, determination of the value of which by the circulatory control inverse model will be explained later. The other resistance coefficients are set to constant values.

14 $${R}_{i}={R}_{i0}\,(i=1,2,3,5,6,7)$$

### Circulatory control inverse model

We explain the models for the no-load stroke volume ratio $a(t)$ and the peripheral vascular resistance ratio $r(t)$ in the followings. Taking into account of the characteristics of the circulatory control system that the ventricular stroke volume increases with increase of the pulse rate^26^, the no-load stroke volume ratio $a(t)$ (see Eq. (6)) is modeled by the interpolation of the linear function $a(t)=b(t)/{b}_{0}\,\,$of the pulse rate $b(t)$ and the constant $a(t)=1$ with a weighting factor ${s}_{a}$, we call it as *stroke volume change rate*.

15 $$a(t)={s}_{a}\frac{\,b(t)}{\,{b}_{0}\,}+(1-{s}_{a})$$

Since the circulatory control system maintains blood pressure constant^24^, and baroreceptors have differential characteristics to effectively respond to short-term changes of blood pressure^25^, the peripheral vascular resistance ratio $r(t)$ (see Eq. (13)) is modeled as the multiplication of the effects of the lower and higher frequency components of the pulse rate variation.

16 $$r(t)={r}_{LF}(t){r}_{HF}(t)$$

where the effect of the lower frequency component of the pulse rate variation ${r}_{LF}(t)$ is modeled by the interpolation of the inversely proportional function ${b}_{0}/{b}_{LF}(t)$ of the lower frequency component of the pulse rate variation ${b}_{LF}(t)$ and the constant 1 with a weighting factor ${s}_{r}$, we call it as *peripheral resistance change rate* whereas the effect of the higher frequency component of the pulse rate variation ${r}_{HF}(t)$ is modeled by the inversely proportional function of the pulse rate.

17 $${r}_{LF}(t)={s}_{r}{b}_{0}/{b}_{LF}(t)+(1-{s}_{r})$$

18 $${r}_{HF}(t)={b}_{LF}(t)/b(t)$$

The lower frequency component of the pulse rate variation ${b}_{LF}(t)$ is expediently modeled by the following second-order low pass filter with the cut-off frequency of ${\omega }_{c}$ and the time constant ${T}_{c}=1/{\omega }_{c}$, we call it *time constant of slow pulse rate variation*.

19 $$\begin{array}{c}\frac{{d}^{2}{b}_{LF}(t)}{d{t}^{2}}+2{\omega }_{c}\frac{d{b}_{LF}(t)}{dt}+{\omega }_{c}^{2}{b}_{LF}(t)={\omega }_{c}^{2}b(t)\\ {b}_{LF}(0)=b(0)\end{array}$$

### Computation

Differential equations for the circulatory dynamics model and the circulatory control inverse model were numerically integrated with the 4-th order Runge-Kutta method. In order to prevent the accumulation of numerical errors, computational results are modified to maintain the total blood volume constant with the interval of one minute of the model time.

### Subjects

The subjects were a healthy male volunteer of 60 s (subject 1), three of 20 s and one of 40 s (subjects 2–5). Informed consent was obtained from the subjects. The study was approved by the Ethics Committee of Graduate School of Engineering, Tohoku University (15A-9). All research methods were performed in accordance with relevant guidelines and regulations.

### Verification experiments

As the input of the present model, the pulse rate was measured for the subjects by a commercially available wearable device (Wristable GPS, SF-810, EPSON, Japan) with the measurement interval of one second in four days (subject 1) with random intervals of 27 months, 3 days, and 3 months, or in one day (subjects 2–5). As the purpose of comparison, systolic and diastolic pressures and pulse rate were also measured in sitting position with an automatic sphygmomanometer (HEM-1025, OMRON, Japan) in the above-mentioned four days with the interval of 30 min (wake up hours) or 60 min (sleeping hours) for subject 1 or in wake up hours with the interval of 30 min in one day for subjects 2–5. Differential equations of the present model were integrated using the measurement data of the pulse rate by the wearable device. The computational time step was fixed to Δ*t* = 0.0002 s according to preliminary calculations. Calculation was performed by a server (HPCT W215s, Intel Xeon Gold 6132, 2.6 GHz 14 Core × 2, 192 GB memory, HPC Tec, Japan) with a typical computational time of 370 s for a 24 hour calculation. Values of the model parameters were determined for each day by comparing the half (subject 1) or all (subjects 2–5) of the sphygmomanometer measurements and the corresponding computations obtained with various combinations of these parameters. The validity of the present DCBP estimation method was then examined by comparing the other half (subject 1) or all (subjects 2–5) of measurements and those of DCBP computations with the determined parameters.

### Determination of the optimum model parameters

Parameters affecting the systemic arterial blood pressure ${P}_{3}(t)$ in this circulatory dynamics model is the standard pulse rate ${b}_{0}$, the elasticity ${E}_{30}$ and the peripheral resistance coefficient ${R}_{40}$ of the lumped systemic arteries at the standard pulse rate. Parameters of the circulatory control inverse model are the stroke volume change rate ${s}_{a}$, the peripheral resistance change rate ${s}_{r}$, and time constant of the slow pulse rate variation ${T}_{c}$. The results of odd number (subject 1) or all (subjects 2–5) measurements in 24 hour period for the systolic and diastolic pressures and the pulse rate, ${P}_{sysm}({t}_{n}),\,{P}_{diam}({t}_{n}),\,{b}_{m}({t}_{n})$ and quantities derived from these measurement results for the average pressure and the pulse pressure, ${P}_{avem}({t}_{n})=({P}_{sysm}({t}_{n})+{P}_{diam}({t}_{n}))/2$, ${P}_{pulsem}({t}_{n})={P}_{sysm}({t}_{n})-{P}_{diam}({t}_{n})$, were used to determine above-mentioned six parameters of the present circulatory system model for each day by comparing them with the corresponding results of calculation, ${P}_{sysc}({t}_{n}),\,{P}_{diac}({t}_{n}),\,{b}_{c}({t}_{n})$, ${P}_{avec}({t}_{n})$, and ${P}_{pulsec}({t}_{n})$ in the following five conditions whereas the results of the even number (subject 1) or all (subjects 2–5) measurements were used to verify the validity of the present method.

1. ${b}_{0}$ is expediently determined as the average value of all the measurement data of the wearable device in 24 hours.
2. ${E}_{30}$ and ${R}_{40}$ are determined so that the average values of the computation for ${P}_{pulsec}({t}_{n})$ and ${P}_{avec}({t}_{n})$ are the same as the corresponding results of the measurement.
3. ${s}_{a}$ and ${s}_{r}$ are determined by multiple conditions that the standard deviation of ${P}_{pulsec}({t}_{n})$ and that of ${P}_{avec}({t}_{n})$ are the same as the corresponding results of the measurement and that the mean square error of ${P}_{pulsec}({t}_{n})$ and that of ${P}_{avec}({t}_{n})$ are the minimum.
4. ${T}_{c}$ is determined so that the mean absolute error of ${P}_{sysc}({t}_{n})$ and ${P}_{diac}({t}_{n})$ is small and the variations of the computed 24 hour blood pressure properly represent the characteristics of the measurement.

According to the present model, increase in ${E}_{30}$ and that in ${R}_{40}$ result in the increase in the mean value of ${P}_{pulsec}({t}_{n})$ and that of ${P}_{avec}({t}_{n})$, respectively, relating to the condition (2). Furthermore, increase in ${s}_{a}$ and that in ${s}_{r}$ result in the increase in the standard deviation of ${P}_{pulsec}({t}_{n})$ and that of ${P}_{avec}({t}_{n})$, respectively, relating to the condition (3). In the parameter determination, a fixed point iterative method was used for the condition (2), and a round-robin method for the conditions (3) and (4).

From Eqs. (5) and (11), we obtain the approximate expressions of ${E}_{30}$ and ${R}_{40}$ by measurement values, ${P}_{sysm}$, ${P}_{diam}$, and ${b}_{m}$; and the stroke volume Δ*V*.

20 $$\begin{array}{c}{E}_{30}=({P}_{sysm}-{P}_{diam})/\varDelta V\\ {R}_{40}=60({P}_{sysm}+{P}_{diam})/(2\varDelta V{b}_{m})\end{array}$$

Values of the other model parameters were determined in reference to the literature^28^ since their effect on the systemic arterial pressure is relatively low.

## Data Availability

The datasets generated during the current study are available from the corresponding author on reasonable request.

## Code availability

The codes used during the current study are available from the corresponding author on reasonable request.
